# Theiler’s virus-induced demyelinating disease as an infectious model of progressive multiple sclerosis

**DOI:** 10.3389/fnmol.2022.1019799

**Published:** 2022-10-13

**Authors:** Steven C. Pike, Nora Welsh, Michael Linzey, Francesca Gilli

**Affiliations:** ^1^Department of Neurology, Dartmouth Hitchcock Medical Center and Geisel School of Medicine, Lebanon, NH, United States; ^2^Integrative Neuroscience at Dartmouth, Dartmouth College, Hanover, NH, United States

**Keywords:** TMEV-IDD, Theiler’s, demyelinating disease, viral MS model, multiple sclerosis

## Abstract

Multiple sclerosis (MS) is a neuroinflammatory and neurodegenerative disease of unknown etiology. However, several studies suggest that infectious agents, e.g., Human Herpes Viruses (HHV), may be involved in triggering the disease. Molecular mimicry, bystander effect, and epitope spreading are three mechanisms that can initiate immunoreactivity leading to CNS autoimmunity in MS. Theiler’s murine encephalomyelitis virus (TMEV)-induced demyelinating disease (TMEV-IDD) is a pre-clinical model of MS in which intracerebral inoculation of TMEV results in a CNS autoimmune disease that causes demyelination, neuroaxonal damage, and progressive clinical disability. Given the spectra of different murine models used to study MS, this review highlights why TMEV-IDD represents a valuable tool for testing the viral hypotheses of MS. We initially describe how the main mechanisms of CNS autoimmunity have been identified across both MS and TMEV-IDD etiology. Next, we discuss how adaptive, innate, and CNS resident immune cells contribute to TMEV-IDD immunopathology and how this relates to MS. Lastly, we highlight the sexual dimorphism observed in TMEV-IDD and MS and how this may be tied to sexually dimorphic responses to viral infections. In summary, TMEV-IDD is an underutilized murine model that recapitulates many unique aspects of MS; as we learn more about the nature of viral infections in MS, TMEV-IDD will be critical in testing the future therapeutics that aim to intervene with disease onset and progression.

## Introduction: Evidence for the role of a virus in multiple sclerosis

Multiple Sclerosis (MS) is an inflammatory and neurodegenerative disease that originates in the central nervous system (CNS). Its etiology remains highly enigmatic, with insufficient evidence on the disease’s exact cause. Many studies have highlighted the role of different environmental and genetic factors in its etiopathogenesis, each adding a new wedge to the MS puzzle and making it a multifactorial and polygenic disease.

Viruses have long been suspected of playing a critical role in the development and progression of MS. Data from epidemiological studies analyzing the geographical, socioeconomic, and genetic features of the disease suggest that exposure to an infectious agent may indeed be involved in triggering the disease (Steiner et al., 2007; Alenda et al., 2014; Virtanen et al., 2014; Tarlinton et al., 2020; Jons et al., 2021; Meier et al., 2021; Xu et al., 2021a; Bjornevik et al., 2022). Several viruses have been studied in association with MS, ultimately speculating a potential pathogenic role for Human Herpes Viruses (HHV), such as varicella-zoster virus (VZV) (Steiner et al., 2007; Jons et al., 2021; Perez-Saldivar et al., 2021), herpes simplex virus 1 (HSV-1) (Steiner et al., 2007; Xu et al., 2021a), HHV-6 (Simpson et al., 2012; Alenda et al., 2014; Virtanen et al., 2014; Meier et al., 2021) and Epstein-Barr virus (EBV) (Virtanen et al., 2014; Guan et al., 2019; Jons et al., 2021; Meier et al., 2021; Perez-Saldivar et al., 2021; Bjornevik et al., 2022). Interestingly, all these viruses are persistent and cause life-long infections with “cellular stress-triggered” reactivation cycles that may be associated with the relapsing-remitting nature of MS (Guan et al., 2019; Tarlinton et al., 2020).

**FIGURE 1:**
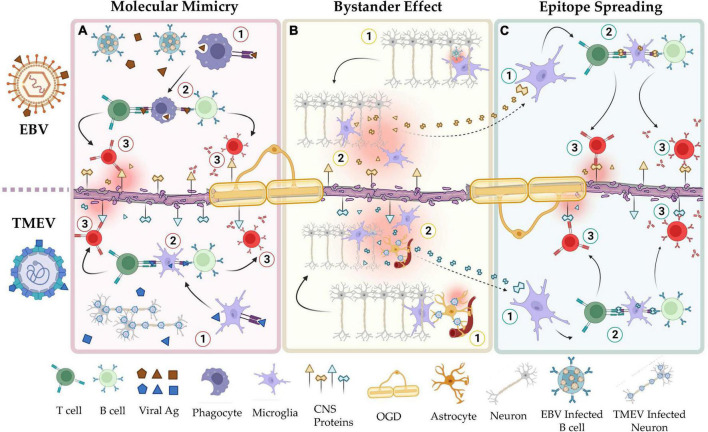
Shared mechanisms of autoimmunity in the viral hypotheses of MS and the TMEV-IDD model of MS. **(A)** Mechanisms of molecular mimicry in EBV (top) and TMEV infection (bottom). Viral peptides, e.g., EBNA-1, share structural homology with CNS resident self-peptides, e.g., GlialCAM. Natural immunization to these viruses induces autoimmunity toward the CNS resident proteins. **(B)** Mechanisms of bystander effect in EBV (top) and TMEV infection (bottom). Responses by resident immune cells to latent or reactivated virus in the CNS induces collateral damage to adjacent tissues. **(C)** Mechanisms of epitope spreading in EBV (top) and TMEV infection (bottom). Peptides released by apoptotic or damaged cells are used to activate self-reactive lymphocytes. In a positive feedback cycle, the autoimmune reaction by these cells releases additional cellular debris. As this cycle continues, the set of self-peptides that are recognized by auto-reactive lymphocytes increases via clonal expansion. This figure was illustrated using Biorender.com.

Molecular mimicry, bystander activation, and epitope spreading have been proposed to potentially explain the viral pathogenesis of MS mediated by HHV (Figure 1; Smatti et al., 2019; Donati, 2020). HHV-6, for example, may play a role in MS through a mechanism of molecular mimicry. Studies have shown a high sequence identity between the U24 gene in the HHV-6 genome and the myelin basic protein (MBP) (Tejada-Simon et al., 2003; Tait and Straus, 2008) increasing the risk of autoimmunity in the CNS. In line with this observation, researchers found, in MS patients, increased T- and B-cell dependent immune responses against this specific antigenic sequence (Tejada-Simon et al., 2003). Furthermore, the theory of cytomegalovirus (CMV)-induced autoimmunity in organ transplant recipients is that cell surface proteins from infected tissues, e.g., CD13, are incorporated into the viral envelope of the virus, severely affecting the allograft function in these patients (Naucler et al., 1996; Rojas et al., 2018). Similarly, it may be speculated that myelin proteins from infected oligodendrocytes could be incorporated into the HHV-6 envelope, inducing an autoimmune reaction in the CNS.

Bystander activation implies that a viral infection can elicit an over-reactive inflammatory response due to persistent infection, tissue damage, and consequent unveiling and presentation of hidden autoantigens. These events can lead to the presentation of autoantigens to autoreactive T and B cells (Figure 1). α-herpesviruses such as VZV and HSV-1 persist in the CNS by establishing a latent infection (Antinone and Smith, 2010; Gilden et al., 2015). Continuous immune control is essential to keep these viruses in latency (Khanna et al., 2004; Cunningham et al., 2006). However, it may still trigger auto-reactive T and B cells bystander activation in susceptible individuals. Overall, this autoimmune response damages myelin-producing cells, causing a release of myelin fragments in the inflammatory environment and triggering a self-sustained breakdown of myelin with the recognition of new subdominant self−epitopes, in a process known as epitope spreading (Figure 1; Lehmann et al., 1992; Cusick et al., 2013). Epitope spreading usually occurs in the context of chronic inflammation and destruction of the target tissue promoting the spreading of the immune response from one autoantigenic determinant to other epitopes not previously recognized by the immune system (Miller et al., 1997; Vanderlugt and Miller, 2002). Disease progression is thus exacerbated by epitope spreading to self−myelin epitopes.

EBV is a γ-herpesvirus that infects more than 95% of the global population (Balfour et al., 2013; Dowd et al., 2013; Winter et al., 2019). Its latent infection is life-long and is established in quiescent memory B cells, presumably by undergoing the germinal center reaction alongside maturing, uninfected B cells (Thorley-Lawson et al., 2013). As a persistent and frequently reactivating virus with major immunogenic influences and a clear epidemiological association with MS (Thacker et al., 2006; Bjornevik et al., 2022), EBV is currently considered to play a leading role in MS pathogenesis, triggering localized inflammation in the CNS. The persistent presence of the virus in the CNS likely provides the signal for initiating and perpetuating inflammation with a consequent inflammation-induced bystander CNS damage mediated by infiltrating B- and T-cells as well as resident astrocytes and microglial cells. On the other hand, molecular mimicry has been reported between the viral protein Epstein–Barr nuclear antigen 1 (EBNA1) and various CNS proteins, including GlialCAM, an immunoglobulin-like cell adhesion molecule expressed in glial cells of the CNS (Lanz et al., 2022), and Anoctamin 2 (ANO1), a voltage-gated calcium-activated anion channel (Tengvall et al., 2019). Upon their activation, “peptides mimic” specific T and B cells can cross-react with self-epitopes, thus leading to tissue pathology, i.e., autoimmunity. Through epitope spreading, these responses may target additional self-epitopes, eventually leading to the accrual of antibody specificity (James et al., 1995; Jog and James, 2020).

Altogether these findings suggest that MS might develop in susceptible individuals due to an alteration of an HHV–host homeostasis that usually ensures a life-long coexistence under continuous immune surveillance in healthy virus carriers. However, despite many years of research and compelling indirect evidence, the role of infectious and viral agents in MS etiology and pathogenesis is still under debate. Research on well-characterized and controlled viral animal models of MS remains an essential step in studying the viral pathogenesis of MS.

## Theiler’s murine encephalomyelitis virus-induced demyelinating diseases as a relevant infectious animal model for multiple sclerosis

Animal models for MS have greatly improved our understanding of the cause and progression of MS. They have proven to be a valuable tool for discovering therapeutic targets and testing their safety and efficacy. However, until recently, pre-clinical studies in animal models of MS took mainly two avenues: one focusing on a purely autoimmune component of the disease and one focusing on the mechanisms of demyelination independent of inflammatory reactions. Neither of these avenues provides clues about the actual triggering of the condition in MS patients.

The experimental autoimmune encephalomyelitis (EAE) series of MS models are mainly used to gain insights into the autoimmune pathogenesis of the disease. In brief, EAE is induced via a peripheral exposure to myelin-specific proteins, e.g., proteolipid protein (PLP), myelin oligodendrocytes glycoprotein (MOG), and MBP, or via adoptive transfer of T cells targeted to these proteins (Lassmann and Bradl, 2017; Glatigny and Bettelli, 2018). Following immunization, autoreactive T cells become active in the secondary lymphoid organs and access the CNS parenchyma through a compromised blood-brain barrier (BBB), subsequently inducing neuroinflammation and demyelination (Lassmann and Bradl, 2017; Glatigny and Bettelli, 2018). Thus, EAE represents a valuable model for studying the development of relapses and acute (neuro)inflammation.

On the other hand, mice treated with toxic substances like cuprizone, lysolecithin, or ethidium bromide represent reliable models for inducing and examining demyelinating/remyelinating events (Lassmann and Bradl, 2017). When exposed to the toxicant, these mice exhibit extensive reactive gliosis, activation of microglia, oligodendrocyte apoptosis, and subsequent reversible demyelination. Spontaneous remyelination is immediately observed after withdrawal of the toxic exposure (Lassmann and Bradl, 2017).

The pathogenic complexity of MS is not easily replicated in both EAE and toxic models. This can be somewhat overcome with the use of infectious models of MS to attempt to replicate the potential viral etiology of the disease.

The study of viral models of MS is not novel (Lassmann and Bradl, 2017). However, given the increasing evidence supporting a viral etiology of the disease, these models have become increasingly popular (and accepted) in recent years. The most common animal used in viral infection models for MS is the mouse (*Mus musculus*), whose CNS can be infected with three different neurotropic viruses, Theiler’s murine encephalomyelitis virus (TMEV), mouse hepatitis virus (MHV), and Semliki Forest virus (SFV), to induce three clinically distinct inflammatory demyelinating diseases all recapitulating different features of MS (Lassmann and Bradl, 2017). Among the three, the TMEV-induced demyelinating disease (TMEV-IDD) is more commonly used to study the viral hypothesis of MS since the other models are much shorter in duration and do not replicate the chronic nature of MS (Mokhtarian et al., 2003; Das Sarma, 2010).

TMEV is a natural single-stranded RNA murine *cardiovirus* of the *Picornaviridae* family that infects the gastrointestinal tract; however, intracerebral injection of this virus leads to varying neuropathology (Olitsky, 1940; Liang et al., 2008). TMEV strains are categorized into two neurovirulence groups (Lorch et al., 1981): (1) highly neurovirulent strains, also known as the GDVII subgroup, including GDVII and FA viruses which cause a rapidly fatal encephalomyelitis in mice (Theiler and Gard, 1940), and (2) low neurovirulence strains, also known as TO subgroup, including the BeAn and DA viruses which produce acute poliomyelitis followed by a chronic demyelinating disease (Lipton, 1975). The latter strains are used to induce TMEV-IDD as a model of progressive MS (PMS).

The type and severity of the disease course are variable across mouse strains, mainly affected by variations in the mouse genome, such as those found in the major histocompatibility complex (MHC) regions associated with susceptibility to the virus (Melvold et al., 1987; Perez Gomez et al., 2021). For example, highly susceptible mouse strains such as *SJL* and *FVB* succumb to TMEV-IDD, whereas non-susceptible strains such as *C57BL/6* or partially susceptible strains such as *C3H* mice develop alternative pathology like epilepsy or myocarditis (Lipton and Dal Canto, 1979; Gómez et al., 1996; Omura et al., 2018). Distinct strains of TMEV can also induce subtle differences in the pathology and disease course. In *SJL* mice, for example, the DA strain has a higher propensity to induce demyelination, while the BeAn strain results in more robust antibody responses (Zoecklein et al., 2003).

TMEV-IDD is clinically described as a biphasic disease; the first stage is an acute polioencephalomyelitis which manifests within the first few weeks post-infection, followed by a chronic progressive demyelinating phase, starting about 1 month after infection and progressing throughout the animal’s lifespan (Lipton, 1975). During the first acute phase, TMEV predominantly infects neurons near the site of infection, spreading then down to the spinal cord (Stewart et al., 2010; Kummerfeld et al., 2012). Like EAE, TMEV-IDD pathology is most prevalent in the spinal cord. Here, leukocytes, predominantly virus-specific T cells, are rapidly recruited from the periphery (Pope et al., 1996; Tsunoda et al., 2002; Kang et al., 2020). As the disease becomes chronic, TMEV infection persists mainly in astrocytes, which are resistant to TMEV-induced apoptosis, whereas neurons and oligodendrocytes are not (Tsunoda et al., 1997; Gerhauser et al., 2018). Interestingly, the BBB is intact during the chronic phase, suggesting that any T and B cell activity is maintained locally (DiSano et al., 2019a,b). Like in PMS, meningeal lymphoid structures form in TMEV-IDD and are theorized to be the source of local B cell proliferation and intrathecal antibody production (Magliozzi et al., 2007; Pachner et al., 2007a; DiSano et al., 2019b).

**FIGURE 2:**
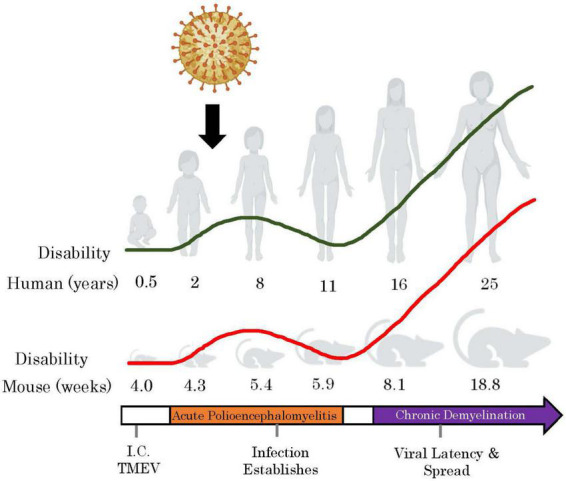
TMEV-IDD models how childhood exposures to common viruses may induce autoimmunity upon adulthood. By mapping the ages of mice in the TMEV-IDD model to human correlates (Wang et al., 2020), we observe that the acute phase of the TMEV infection occurs during childhood, and the chronic phase begins in early adulthood. Red line represents relative clinical disability accumulation in TMEV-IDD over time and the green line shows this with respect to human years. I.C, intracranial. This figure was illustrated using Biorender.com.

TMEV-IDD etiology parallels well the viral hypothesis of MS (Figure 2). In TMEV-IDD, the early viral infection causes vast neuronal and oligodendroglia apoptosis. These events release myelin and neuronal antigen, which are thought to initiate an autoimmune reaction. The proposed mechanisms by which TMEV may induce autoimmunity are like those that aim to explain the viral pathogenesis of MS: bystander activation, epitope spreading, and even molecular mimicry.

The bystander effect is possibly the most apparent mechanism by which TMEV may induce autoimmunity (Figure 1). CNS resident antigen-presenting cells (APCs) present both viral and myelin epitopes during the chronic stage of the disease but only viral epitopes during the acute phase (Katz-Levy et al., 1999, 2000). This suggests that the immune response to the virus results in inflammatory signaling that allows microglia and/or other APCs to present self-antigen to autoreactive lymphocytes. In addition, microglia infected with TMEV can release viral RNA via exosomes, stimulating distal, uninfected cells to become pro-inflammatory (Luong and Olson, 2021).

Researchers have also detected epitope spreading during TMEV-IDD (Figure 1). By mapping the expansion of autoreactive T cell clones throughout the disease course, it was demonstrated that there is a particular order in which the epitopes spread: in wild-type TMEV infections, the earliest T cell clones are PLP_139–151_ specific, followed by PLP_178–191_, PLP_56–70_, and finally MOG_92–106_ (Miller et al., 1997). This exemplifies how the initial immunity toward a single fragment can result in autoimmunity to other fragments of the same molecule (other PLP fragments) or molecules (MOG).

Lastly, TMEV has been used to model viral molecular mimicry (Figure 1). Although wild-type TMEV does not seem to have direct cross-reactivity with self-peptides, epitopes such as PLP_139–151_ have been genetically engineered into wild-type TMEV to study this phenomenon (Croxford et al., 2002). These strains induce a much faster disease with earlier CD4 + T cell responses compared to the wild-type virus. In addition, other genetically engineered TMEV strains have been constructed with sequences of *Haemophilus influenzae* virus (HI574-598), a natural high-homology mimic of PLP (Croxford et al., 2002). This strain also produced a faster disease than wild-type TMEV but slower than the PLP-TMEV.

Interestingly, autoimmunity via the HI574–598 fragment is only possible via a virus (Carrizosa et al., 1998). Although immunization to this fragment alone results in expanded T cell populations that cross-react with PLP, they lack Th1 differentiation. The fact that Th1 differentiated cells are required for the disease induction suggests that a particular cytokine environment is needed to establish autoimmunity, in addition to the presentation of self-antigen.

Although there are many strengths to using TMEV-IDD as a model the viral hypothesis of MS there are some limitations. Firstly, we do not observe relapses and remissions with TMEV-IDD, but rather, it follows a disease course more like that of primary progressive MS. In addition, TMEV-IDD, like EAE, is primarily a spinal cord disease and fails to replicate the level of cerebral damage as we see in MS. Lastly, TMEV is very different in structure and tropism from the HHVs that are linked to MS and may illicit different immunological responses. Given that all mouse models have their strengths and weaknesses, TMEV-IDD is the best available option when studying how viruses can progress to a MS-like disease.

## Neuro-immune response in Theiler’s murine encephalomyelitis virus-induced demyelinating diseases

The inflammatory response to viral infection in humans is a dynamic process with complex cell interactions led by the immune system and influenced by both host and viral factors. Due to this complexity, the relative contributions of each factor in shaping the inflammatory response can be studied in animal models. Most viral infections are initially controlled by different innate immune system elements, including phagocytic cells, e.g., microglia, interferons and interferon-stimulated genes (ISGs), and the CS (Chhatbar and Prinz, 2021). If viral replication outpaces innate defenses, a specific adaptive immune response, consisting of B and T lymphocytes, is mobilized.

Following direct CNS infection, TMEV persists in microglia and leads to developing a chronic progressive demyelinating disease associated with innate and adaptive inflammation in the CNS (Tsunoda and Fujinami, 2010). As such, TMEV-IDD is used as a powerful model to examine changes in the innate and adaptive immune responses related to a persistent infection of the CNS leading to demyelination and disability progression.

### Role of B cells

MS has historically been viewed as a T cell-mediated autoimmune disease partly because of the T cell-specific pathology observed and studied in the MS murine model EAE (Weiner, 2004). However, the recent and successful use of B-cell depleting therapies (BCDT) reveals a previously unrecognized critical role for B cells in MS. On the other hand, B cells have been implicated with MS since 1942, when increased levels of immunoglobulins (Igs) in the cerebrospinal fluid (CSF) of patients were first described to be suggestive of MS (Kabat et al., 1942). More recent studies also indicate that intrathecal synthesis of Igs correlates with MS disease progression (Joseph et al., 2009; Farina et al., 2017; Ozakbas et al., 2017). Antibody-secreting cells (ASC) in the CNS may be pathogenic either by the direct action of reactive Igs or locally secreting neurotoxic products (Michel et al., 2015; Jain and Yong, 2021). Substantial proof for the involvement of B cells in MS has also come from the finding of ectopic B cell-rich lymphoid-like follicles (ELF) in the meninges of patients with PMS (Magliozzi et al., 2007; Reali et al., 2020). These ELF correlate strongly with cortical pathology and disease severity (Reali et al., 2020).

In the context of a viral etiology for MS, it is noteworthy that humoral responses within the CNS are prevalent in neurotropic viral infections, with ASC contributing to local protection. In MS patients, intrathecal synthesis of Igs was detected against measles, rubella, varicella zoster, mumps and EBV, and in most cases, antibodies against more than one virus are present (Arnadottir et al., 1979; Sindic et al., 1994; Kakalacheva et al., 2011). In contrast, antibodies against non-neurotropic viruses, e.g., CMV, are rarely detected in the CSF from MS patients (Sindic et al., 1994).

TMEV-IDD provides an excellent model to interrogate the function of B cells in a viral model of MS (DiSano et al., 2019b). Increased concentrations of intrathecally produced Igs and substantial infiltration of ASC and B memory cells are observed in the spinal cord and the meninges of persistently infected mice, suggesting a potentially critical role for Igs and B cells in the chronic progressive phase of the demyelinating disease (Pachner et al., 2007a,b; DiSano et al., 2019a,b; Jin et al., 2020).

Many of the intrathecal Igs in TMEV-IDD are reactive against the virus (Pachner et al., 2007b; Tsunoda and Fujinami, 2010). However, mice chronically infected with TMEV also produced antibodies to self-antigen within the CNS, primarily in response to damaged tissue and the consequent release of CNS unique antigens (Jin et al., 2020). These autoantibodies target multiple self-antigens (because of epitope spreading) and are independent of the initial trigger once tolerance is broken (Figure 1).

A recent finding in TMEV-IDD also described the development of B cell-rich meningeal aggregates, like those observed in patients with MS (DiSano et al., 2019b,^2020^). Although these aggregates in TMEV-IDD do not fully develop into EFLs, like in MS, they correlate strongly with pathology and disease severity (DiSano et al., 2019b).

Overall, these findings in TMEV-IDD demonstrate that the model well recapitulates B cell-specific disease phenotypes that are also observed in patients with MS. Therefore, these findings support TMEV-IDD as a valuable model for investigating the role of B cells in a chronic neuroinflammatory condition and test new potential therapeutic strategies.

#### B cell-depleting therapies in Theiler’s murine encephalomyelitis virus-induced demyelinating diseases

BCDT efficiently suppresses acute inflammatory disease activity in relapsing-remitting MS and may slow progression in a subgroup of primary-progressive MS patients (Bar-Or et al., 2008; Montalban et al., 2017). Considering the increasing interest in these treatments in MS, the susceptibility of B cells to TMEV infection is particularly important. However, studies in our laboratory showed that anti-CD20 BCDT administered to TMEV-DD mice worsened rather than improved the overall disease course (Gilli et al., 2015). In these mice, systemic and CNS antibody responses were suppressed during the treatment. Higher viral loads were detected in treated mice vs. controls, and the viral levels correlated negatively with IgG production in the brain (Gilli et al., 2015).

BCDT causes significant worsening of the early encephalitis and faster progression of disability, as well as exacerbation of the pathology at the end stage of the disease (Gilli et al., 2015). Interestingly, a similar severe acute disease worsening was also seen in the MOG_33–35_ EAE model when treated with BCDT (Matsushita et al., 2008; Weber et al., 2010). This was thought to be due to heightened inflammation caused by removing IL-10-producing CD20 + regulatory B10 cells in EAE. The similar timing and exacerbation of disease symptoms in TMEV-IDD and EAE studies suggest that a similar B10 mechanism may be operative in both models. The fact that mice had worse neurological function when CNS antibody production was reduced is consistent with a protective role for CNS IgG. Protection could be accomplished by suppression of viral replication (Stohlman et al., 2002; Metcalf and Griffin, 2011), clearance of myelin debris (Vargas et al., 2010), promotion of remyelination (Asakura and Rodriguez, 1998), or other mechanisms. This positive role for some populations of locally produced IgG does not rule out pathological effects for different populations of CNS IgG. These findings have considerable relevance to the use of BCDT in MS. Patients with MS receiving BCDT may develop highly inflammatory syndromes because of the absence of B regulatory cells or worsened CNS or systemic infections because of the inability to mount a solid anti-pathogen humoral immune response.

### The innate immune system

According to evidence from the recent decade, the innate immune system plays a critical role in the initiation and progression of MS. This system activates and regulates the effector functions of T and B cells, similar to what happens during infections (Sospedra and Martin, 2005; Weiner, 2008; Gandhi et al., 2010). In the CNS, such modulation is mainly mediated by resident immune cells, i.e., microglia and astrocytes, and locally produced inflammatory proteins, like the interferons and the complement system (CS) (Ingram et al., 2014; Liddelow et al., 2020; Absinta et al., 2021).

TMEV-IDD is an appropriate model to study innate immunity to viral pathogens in the CNS, given the critical role that this arm of the immune system plays in the pathogenesis of the demyelinating disease. As stated above, clinically, TMEV-IDD is considered a biphasic disease (Lipton, 1975). Nevertheless, its immunopathogenesis can be divided into not just two but three discrete stages, two of which mainly involve the innate immune system (Sospedra and Martin, 2005; Gilli et al., 2016). Following TMEV infection, the innate immune response is immediately activated in the CNS (Olson et al., 2001; Olson and Miller, 2009), thereby providing an adjuvant signal for the induction of naive and memory virus-specific adaptive immune responses (stage 1, polioencephalomyelitis). A prolonged adaptive inflammatory reaction, which involves both T and B cells, determines the development of the demyelinating disease (stage 2). Finally, there is a reversion to a chronic innate-like immune response associated with extensive CNS damage and progressive neurodegeneration (stage 3) (Gilli et al., 2016). This last stage is characterized by an upregulation in the CNS of almost all innate immune pathways, including toll-like receptor (TLR)-signaling pathways, e.g., TLR7, TLR8, TLR9, and type I Interferon responses (Ulrich et al., 2010; Gilli et al., 2016).

While triggering these signaling pathways may be beneficial during acute infections, persistent activation of the innate immune system contributes to neurodegeneration (Heneka et al., 2014). Interestingly, a similar multi-stage process is evident in MS, where the relapsing-remitting phase is thought to be mainly driven by the adaptive immune system, while a heightened innate immune response and neurodegeneration primarily characterize the late progressive stage of the disease (Weiner, 2008; De Jager et al., 2009; Kamma et al., 2022).

Another component of the innate immune system critically involved in neurodegenerative processes is the CS. This protein cascade has traditionally been considered a complex innate immune surveillance system, playing a pivotal role in defense against pathogens and host homeostasis (Merle et al., 2015a). The proteins collaborate to opsonize pathogens, enhance inflammatory responses, and induce cytotoxicity, helping the immune cells to fight infections (Merle et al., 2015b). However, in the last few decades, complement activation has been increasingly involved in the progression of neurodegenerative disorders, including MS (Lee et al., 2019; Morgan et al., 2020). Activation of the CS is also involved in the pathogenesis of many antibody-mediated autoimmune diseases (Leffler et al., 2014; Nytrova et al., 2014).

Autoantibodies can directly damage target tissues, and complement activation is a downstream mediator of this injury (Merle et al., 2015a). The classical complement cascade, one of the three activation pathways of the CS, is activated when IgG or IgM are bound to their target antigen on either a pathogen cell membrane or an immune complex (Merle et al., 2015a). Thus, in MS, intrathecally produced IgG and IgM may lead to the activation of the CS, thereby promoting CNS tissue damage. Accordingly, deposition of key complement components and activation products, e.g., C1q, C3, C4b, and the membrane attack complex (MAC), have been detected in MS patient lesions (Ingram et al., 2014; Watkins et al., 2016; Cooze et al., 2022). Complement activation has been demonstrated in acute and chronic MS lesions (Ingram et al., 2014; Watkins et al., 2016).

In TMEV-IDD, activation of the CS is evident at the early and chronic stages of the disease (Libbey et al., 2010; DiSano et al., 2019a; Linzey et al., 2022). In a recent study, the CS, specifically the classical complement pathway, was associated with TMEV-IDD pathogenesis, as the expression of critical complement components like C1q, C3, and C3aR1 were all correlated to a worse disease outcome (Linzey et al., 2022). In line with this finding, C1q and C3 depositions were observed in CNS regions characterized by inflammation, i.e., microglia and astrocytes activation, demyelination, and axonal damage (Linzey et al., 2022). These results reveal an association between TMEV-IDD and activation of the classical complement pathway by antigen-antibody immune complexes that appear to contribute significantly to the disease severity.

Microglia and astrocytes play an essential role in the CNS, contributing to many functions, including homeostasis, immune response, BBB maintenance, and synaptic support (Liddelow et al., 2020). Microglia, the resident phagocytic immune cell of the CNS (Schafer et al., 2012; Favuzzi et al., 2021), are exquisitely sensitive to injury and disease, altering their morphology and phenotype to adopt an “activated” state in response to pathophysiological brain insults. These CNS cells are a culprit of neuroinflammation in several neurodegenerative diseases, including MS (Hickman et al., 2018; Guerrero and Sicotte, 2020; Xu et al., 2021b).

During TMEV-IDD, microglia undergo activation, proliferation, and changes in morphology, with detrimental and beneficial effects (Herder et al., 2015). Activated microglia promote neuronal repair through the secretion of anti-inflammatory growth factors and cytokines. However, activated microglia can also generate secondary neuronal injury via the production of pro-inflammatory cytokines, e.g., type 1 interferons, IL-1, IL-6, IL-23, TNFα, complement components such as C1q (Libbey et al., 2017; Gerhauser et al., 2019), reactive oxygen species (ROS) (Iwahashi et al., 1999), and proteases (Crapser et al., 2021). These MOG drive a CNS compartmentalized inflammation by stimulating local immune cells, a phenotype observed in PMS like in TMEV-IDD (Absinta et al., 2021; Cooze et al., 2022).

Astrocytes are supporting cells within the CNS that have numerous functions, including providing structural support, insulating receptive surfaces, and buffering the extracellular compartment (Santello et al., 2019). During CNS insult or neurodegenerative processes, astrocytes can respond to pathological changes by releasing extracellular MOG, such as neurotrophic factors, e.g., BDNF and VEGF, pro-inflammatory cytokines, e.g., IL-1β, TNF-α, stimulatory MOG like MHC II and CD80/86 (Li et al., 2019; Escartin et al., 2021), inducible nitric oxide synthase (iNOS) and cytotoxins such as Lcn2, through reactive astrogliosis (Li et al., 2019). As a result, like microglia, astrocytes have neuroprotective or neurotoxic effects in the CNS (Linnerbauer et al., 2020).

Astrocytes play multiple roles in the evolution of MS lesions, not only by recruiting lymphocytes and contributing to tissue damage but also by confining inflammation and promoting lesion repair (Zeinstra et al., 2003; Liddelow et al., 2017; Ponath et al., 2018; Li et al., 2019). Likewise, reactive astrocytes have been researched for their role in the disease progression of TMEV-IDD (Allnoch et al., 2019). Astrocytic infection by TMEV stimulates TLR3, which initiates an antiviral response, and increases the production of pro-inflammatory MOG such as TNFα and iNOS (Allnoch et al., 2022). Also, reactive astrocytes have been shown to release, in the CNS, the C3 factor, i.e., the central protein in the CS (Pekna and Pekny, 2021). Accordingly, throughout TMEV-IDD, a significant upregulation of C3 expression and deposition is observed as stimulated by reactive astrocytes (DiSano et al., 2019a; Linzey et al., 2022).

### B cells –astrocytes –microglia axis

The inflamed CNS in MS emerges as a B cell fostering environment, as evidenced by the continuous synthesis of intrathecal Igs and the development of B cell-rich ELFs in chronic progressive patients. In this favorable environment, B cells contribute to developing CNS-compartmentalized inflammation and progressive CNS injury. However, it is still unclear how B cells are fostered within the CNS and how they contribute locally to the propagation of neuroinflammation behind a closed BBB. Interestingly, recent studies have shown a potential bi-directional interaction in the CNS between B cells and glial cells, mainly astrocytes and microglia, which may influence the propagation of the neuroinflammatory stimuli associated with MS progression (Touil et al., 2018; Hollen et al., 2020).

Thus far, TMEV-IDD has provided valuable insights into the relationship between B cells and glial cells. For example, in this model, it has been shown that inflammatory microglia produce a range of soluble factors such as cytokines and complement proteins to induce astrogliosis (Liddelow et al., 2017; Linzey et al., 2022). Following their activation, reactive astrocytes further amplify inflammation by releasing pro-inflammatory and neurotoxic mediators, including the complement factor C3 (Allnoch et al., 2019; DiSano et al., 2019a). C3 is the central protein of the CS, and its hydrolysis leads to the production of anaphylatoxins (C3a and C5a), which stimulate the secretion of various pro-inflammatory cytokines (Werneburg et al., 2020). Moreover, recent work has shown that C3 can interact with B cells to aid their maturation, linking the CS and B cell activity (Rossbacher and Shlomchik, 2003).

Another connection linking CNS resident cells to pro-inflammatory B cell activation is CXCL13 and its receptor CXCR5. CXCL13 is a potent B cell chemoattract that neurons can produce during various insults (Trolese et al., 2020). It has also been shown that the receptor for CXCL13, CXCR5, is expressed by astrocytes, and this interaction promotes the development of reactive astrocytes (Jiang et al., 2016). Once activated by CXCL13, astrocytes secrete essential B cells growth factors such as BAFF and APRIL (Touil et al., 2018; DiSano et al., 2019b), ultimately fostering B cells within the CNS. Interestingly, researchers have reported significantly increased CXCL13, BAFF, and APRIL levels in the CNS of TMEV-IDD (DiSano et al., 2019b; Harrer et al., 2021).

### Sex differences in Theiler’s murine encephalomyelitis virus-induced demyelinating diseases

Biological sex profoundly affects the susceptibility and course of MS, with a higher disease prevalence and overall better prognosis in women than men [for review, see Gilli et al. (2020)]. The risk of developing MS is 3–4 times higher in women (Orton et al., 2006; Harbo et al., 2013). However, while men are less likely to develop the disease, they are at increased risk for worse clinical outcomes, e.g., more significant disability over time (Ribbons et al., 2015), faster time to progression (Jelinek et al., 2016) and overall, more neurodegenerative lesions compared to women (van Walderveen et al., 2001; Pozzilli et al., 2003).

In the context of the viral etiology of MS, it is worth noting that many viral infections manifest sex differences like those observed in MS: longitudinal studies found, for example, a significant difference in EBV and HSV1 prevalence between men and women, showing an overall infection preponderance and higher antibody titers in females than males (Jacobsen and Klein, 2021). On the other hand, the intensity and severity of disease caused by some neurotropic viruses, including EBV and HSV1, are more significant for males than females (Han, 2001; Jean et al., 2007; Balogun et al., 2009; Murphy et al., 2009).

TMEV-IDD is characterized by a similar sexual dimorphism, with female mice showing an overall infection preponderance and higher antibody titers but male mice being more severely affected (Alley et al., 2003; Fuller et al., 2005). Interestingly, different strains of mice exhibit different susceptibility to the development of TMEV-IDD and variations in their sexual dimorphism. In *SJL* mice, for example, females seem slightly more susceptible than males (Hill et al., 1998). Still, males exhibit worsened clinical scores over time as well as moderately more demyelination in the CNS compared to their female counterparts (Fuller et al., 2005). Other studies did not confirm the increased TMEV-IDD prevalence in female *SJL* mice (Alley et al., 2003). However, it is noteworthy that in the *SJL* strain, the analysis may be confounded by the high susceptibility to TMEV-IDD of these mice. Differently from the *SJL* strain, in the *C57L* background, male mice are highly susceptible to TMEV-IDD, whereas females are resistant (Kappel et al., 1990).

Gonadal hormones, especially estrogens and androgens, have long been found to account for some sex differences in MS (Tomassini et al., 2005; Tomassini and Pozzilli, 2009; Ysrraelit and Correale, 2019), although molecular mechanisms mediating these effects remain to be elucidated. Accordingly, in *SJL* mice, at least partly, susceptibility is sex-hormone specific as castrated male mice exhibit increased susceptibility to TMEV infection (Fuller et al., 2007). At the same time, this effect is rescued by estrogen treatment (Fuller et al., 2007). More recently, the effects of sex chromosome genes have also been implicated as contributors in animal models of MS (Smith-Bouvier et al., 2008; Du et al., 2014; Voskuhl et al., 2018), particularly the potentially harmful impact of the second X chromosome in females and/or the unique Y chromosome in males. It is widely speculated that this genetic difference may account for the different prevalence in the incidence of several diseases, including MS.

Sex chromosomes and sex hormones contribute to differential regulation of immune responses between sexes. Since the immune system plays a critical role in the initiation and progression of MS as well as in viral infections, sex-driven differences in the type and magnitude of the inflammatory immune response represent a vital cue. It is well known the immune system displays widespread sexual dimorphism. Generally, females mount stronger humoral and cell-mediated immunity than males (Klein and Flanagan, 2016; Gal-Oz et al., 2019). The weaker male response to viruses may allow the invader to cause more damage, whereas the intense and persistent female response reduces the tissue injury. In line with this observation, data from our laboratory demonstrate that following TMEV infection, female *SJL* mice induce a quicker and stronger peripheral anti-TMEV humoral and cellular immune response (Welsh et al., 2020). This finding is in line with the results of a previous study which showed a higher level of the initial antiviral immune (both humoral and cellular) response in female compared to male mice (Fuller et al., 2005).

Despite this clear distinction of similarities to the sexual differences in MS, very little research has utilized the TMEV-IDD model to study these dimorphisms. Additionally, most of the research in TMEV-IDD has been using female mice only due to the aggressive nature of the *SJL* male mice. The National Institute of Health (NIH) currently expects sex as a biological variable to be factored into research designs, analyses, and reporting in clinical and pre-clinical studies. Given the solid sexual dimorphism reported in TMEV-IDD and the high relevance for MS, appropriate analyses and transparent reporting of data by sex may significantly enhance the rigor and applicability of pre-clinical biomedical research using this model.

## Conclusion

TMEV-IDD represents an accurate model system of MS due to several distinct features. In the first place, TMEV-IDD disability slowly progresses, secondary to chronic demyelination, inflammation, and axonal/neuronal damage. Like MS, in TMEV-IDD, the disease progresses through 20–25% of the lifespan and then usually plateaus (Figure 2). Moreover, this murine model parallels typical MS neurodegenerative hallmarks such as atrophy (Umpierre et al., 2014), neuronal death (Mecha et al., 2013), axonal injury (Tsunoda and Fujinami, 2010), and, most importantly, persistent compartmentalized CNS inflammation (Gilli et al., 2016; DiSano et al., 2019a). In this model, inflammation is often sustained behind a relatively closed BBB (DiSano et al., 2019a), supported by diverse CNS-infiltrating immune cells and CNS-resident cells. TMEV-IDD not only represents an excellent model for the study of the pathogenesis of MS, but it also provides a model for studying disease susceptibility factors, sexual dimorphism, CNS viral persistence, and virus-induced autoimmune disease. This model system provides a solid basis for dissecting cell and molecular mechanisms involved in developing and progressing a virus-induced demyelinating disease and characterizing the efficacy of targeted therapeutics.

## Author contributions

SP, NW, ML, and FG outlined the subject for the review. SP, NW, and ML reviewed the literature and wrote the manuscript. SP drafted the figures. FG edited and revised the manuscript. All authors contributed to the article and approved the submitted version.
